# The Health Benefits of Emodin, a Natural Anthraquinone Derived from Rhubarb—A Summary Update

**DOI:** 10.3390/ijms22179522

**Published:** 2021-09-01

**Authors:** Monika Stompor-Gorący

**Affiliations:** Department of Human Pathophysiology, Institute of Medical Sciences, University of Rzeszów, Warzywna 1a, 35-310 Rzeszów, Poland; monika.stompor@gmail.com

**Keywords:** anthraquinones, emodin, anti-inflammatory properties, anticancer activity, antimicrobial agents

## Abstract

Emodin (6-methyl-1,3,8-trihydroxyanthraquinone) is a naturally occurring anthraquinone derivative found in roots and leaves of various plants, fungi and lichens. For a long time it has been used in traditional Chinese medicine as an active ingredient in herbs. Among other sources, it is isolated from the rhubarb *Rheum palmatum* or tuber fleece-flower *Polygonam multiflorum*. Emodin has a wide range of biological activities, including diuretic, antibacterial, antiulcer, anti-inflammatory, anticancer and antinociceptive. According to the most recent studies, emodin acts as an antimalarial and antiallergic agent, and can also reverse resistance to chemotherapy. In the present work the potential therapeutic role of emodin in treatment of inflammatory diseases, cancers and microbial infections is analysed.

## 1. Introduction

**Figure 1:**
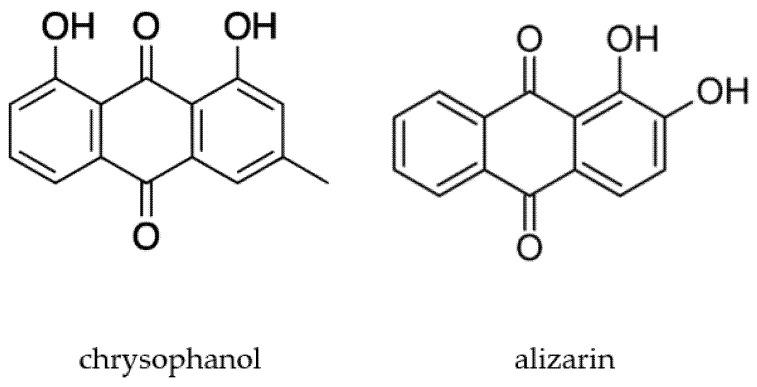
The natural antra-quinones.

Anthra-compounds are anthracene derivatives that have three fused benzene rings and one or more hydroxyl groups, which can bind to sugar molecules. That is why, in nature, they are found in the form of anthraquinone glycosides, most commonly with glucose or rhamnose as the sugar component. They are solid substances, usually in the form of orange crystals. The activity of anthra-compounds depends on their chemical structures and are related to the presence of hydroxyl groups at C-1 and C-8 in the aromatic ring, the nature of a substituent at C-3 and the number of sugar residues. Anthra-compounds occur either in oxidized (anthraquinones) or reduced form (anthrones, anthranols), and also as dimers (dianthrones). Reduction of anthraquinones leads to unstable anthrahydro-quinones and oxyanthrones. Naturally occurring anthraquinones find application as natural dyes, e.g., alizarin (Figure 1). Moreover, they display a range of desired biological activities. For example, one of the natural anthraquinones. chrysophanol, acts as an anti-inflammatory [1] and anticancer [2] agent. Anthraquinone derivatives isolated from microbial cells, such as 1-*O*-methyl chrysopchanol, isolated from *Amycolatopsis thermoflava* SFMA-103, have antihyperglycemic [3] and antitumor [4] properties. Similarly, naphthoquinones (e.g., 7-methyl juglone) and their derivatives have been widely studied with respect to their biological activity [5].

Anthraquinones isolated from roots and leaves of plants belonging to families *Polygonaceae*, *Rhamnaceae*, *Rubiaceae*, *Fabaceae* and *Scrophulariaceae* and from fungi and lichens show a wide range of therapeutic effects [6]. They have antihyperlipidemic, cholesterol-lowering, antiseptic, anticancer [7], and antimicrobial [8,9] properties. They can also find application in the treatment of kidney diseases, such as renal interstitial fibrosis (RIF) [10], or liver dysfunction. Additionally, there is research into new methods of functionalization of anthraquinones, and into synthesis of co-polymeric nanostructures designed for photodynamic therapy [11].

Emodin can be found in the roots, leaves, bark and trunk of several plants, such as *Senna alata* (*Cassia alata*) [12], *Rumex abyssinicus* [13], *Odontites serotina* [14], *Reynoutria japonica* [15], *Polygonum cuspidatum* [16]. The source of emodin in the human diet is rhubarb [17]. The roots and rhizomes of *R. palmatum* contain about 2.31 mg/g of emodin. Furthermore, the ethanol and acetone extract are 5.32 mg/g and 8.04 mg/g, respectively [18].

The objective of this review is to summarize the most recent research on one of the most important anthraquinones of natural origin—emodin. Potential employment of emodin for the treatment of oncological, inflammatory and neurodegenerative diseases and as a natural antimicrobial agent have been described. So as to support future full-profile study on emodin with respect to its clinical use, effects of this anthraquinone derivative on the activity of some other drugs is also presented in this work.

## 2. Pharmacological Effects of Emodin

**Figure 2:**
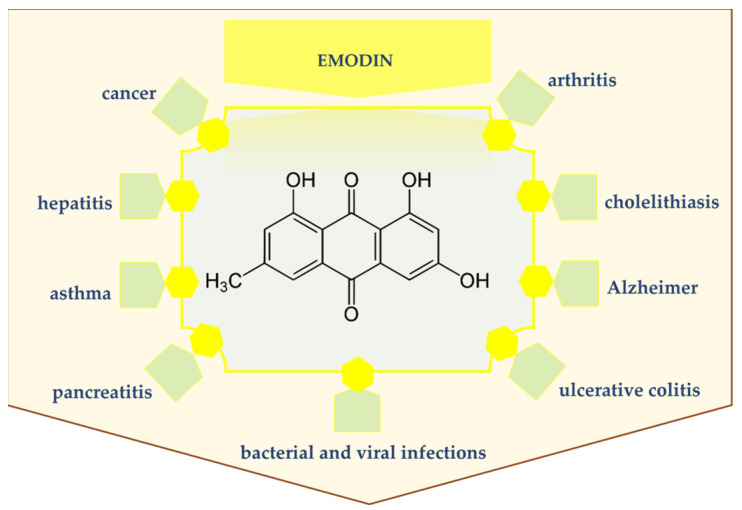
Emodin’s potential in human disease treatment.

Emodin is a natural compound of the anthraquinone family with therapeutic properties (Figure 2). It is an effective inhibitor of many cell signaling pathways. It has been confirmed that emodin inhibits processes of neoplasia at the stages of proliferation, invasion and angiogenesis. Additionally, emodin is used to treat cholelithiasis and hepatitis and to cure bacterial and viral infections. High therapeutic potential of emodin arises from its ability to interact with many molecular targets related to inflammatory and cancer processes [19,20]. In addition, it has been proved that emodin in combination with other biologically active substances, such as baicalin or liquiritin, is an anti-inflammatory agent [21]. What is more, emodin has a therapeutic potential for the treatment of ulcerative colitis [22], keratitis [23], cardiac fibrosis [24] and rheumatoid arthritis [25]. According to the most recent research reports, emodin isolated from *Artemisia annua* has antimalarial properties [26]. In an in vivo study it was demonstrated that emodin acts as an analgesic [27]. Moreover, emodin and artinemol may be effective antiviral drugs for the treatment of patients with COVID-19 [28].

The activity of emodin and its derivatives is related to their influence on inflammatory processes and their immunomodulatory activity [29]. Because of the uncertainty about the safety of using emodin for pharmacotherapy, in vivo studies in mice have been carried out to establish its safe dosage [30]. The results showed that administration of emodin at the doses of 20, 40, and 80 mg/kg for 12 weeks is safe and did not cause any pathophysiological disorders in major organs in mammals.

### 2.1. Anticancer Activity of Emodin

Conventional treatment of cancer, including chemotherapy and radiation therapy are often ineffective, very expensive and associated with many unwanted side effects. Moreover, not all patients benefit from this kind of treatment. More and more often research on development and implementation of alternative, non-toxic drugs and combined therapies involves the use of natural chemotherapeutics, isolated from plant extracts.

**Figure 3:**
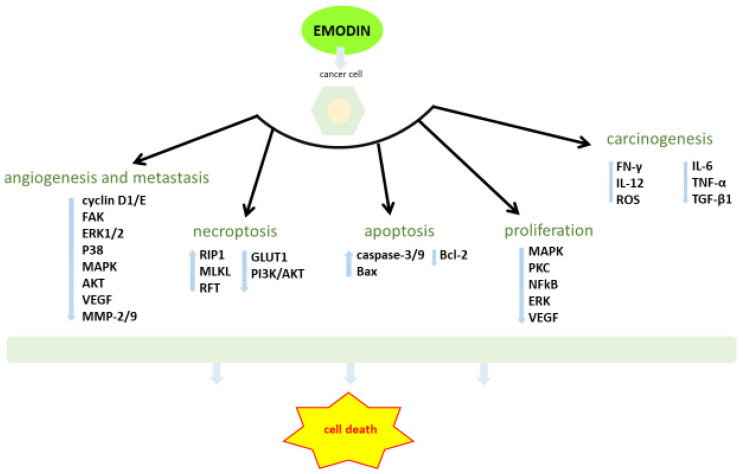
The main anticancer molecular mechanism of emodin. FAK: focal adhesion kinase; ERK1/2: extracellular signal-regulated protein kinase 1/2; MAPK: mitogen-activated protein kinase; AKT: oncogenic protein kinase B; VEGF: vascular endothelial growth factor; MMP: matrix metalloproteinase; RIP1: receptor-interacting protein 1; MLKL: mixed lineage kinase domain like pseudokinase; RFT: fibroblast growth factor 2 (FGF-2) transcription; Bax: bcl-2-like protein 4; Bcl-2: B-cell lymphoma 2; PKC: protein kinase C; NF-κB: nuclear factor kappa-light-chain-enhancer of activated B cells.

Emodin suppresses the development of many types of cancer [31,32,33,34,35] by regulating expression of genes associated with carcinogenesis, apoptosis, proliferation, invasion and metastasis of cancer cells (Figure 3). Dumit et al. [36] determined the effects of emodin on the redox state of cells and on mitochondrial homeostasis. They found that cells with efficient respiratory metabolism are less susceptible to emodin, whereas cells under glycolytic metabolism are more vulnerable to this compound. They also documented that emodin causes oxidative stress that particularly disturbs cancer cells.

Li et al. [37] reported that emodin selectively suppresses N2 neutrophils to prevent hypercoagulation and lung carcinogenesis. Emodin treatment resulted in ameliorated hypercoagulation and lung carcinogenesis accompanied by decreased N2 neutrophils (CD66b+) in the alveolar cavity. Enzyme-linked immunosorbent assay (ELISA) showed that there were more interferon gamma (IFN-γ), interleukin (IL)-12 and reactive oxygen species (ROS) and less IL-6, tumor necrosis factor alpha (TNF-α) and transforming growth factor beta1 (TGF-β1) in the alveolar cavity in the emodin group than those in the control group. In vitro, emodin at the dose of 20 μM had no effect on cell viability in HL-60N1, but increased ROS and decreased autophagy, and thus induced apoptosis in HL-60N2 with these morphological changes. The most recent research results indicate that emodin regulates cell cycle in non-small lung cancer (NSCLC) through hyaluronan synthase 2 (HA2)-HA-CD44/receptor for the hyaluronic acid-mediated motility (RHAMM) interaction-dependent signaling pathway [38]. Li et al. [39] synthesized new emodin derivatives, which were further tested for antitumor activity against HCT116 cancer cells.

#### 2.1.1. Proliferation

The effect of emodin on cell proliferation was studied and confirmed for many cancer cell lines, including breast [40], skin [41], pancreas [42], colon [43,44], ovarian [45], prostate [46], esophageal [47], bladder [48], and endometrial [49]. For example, the IC_50_ values for MCF-7 (breast) and HepG2 (liver) cancer cells were 52.72 µM and 43.87 µM, respectively [50]. There are also known efficient antiproliferative substances obtained by chemical modifications of emodin [51,52]. Novel emodin derivatives often have higher antiproliferative activity against cancer cells, such as HT-29, PC-3 or HeLa [53], than emodin.

Antitumor properties of emodin are associated with inhibiting the activity of tyrosine kinases, such as mitogen-activated protein kinase (MAPK), protein kinase C (PKC), factor kappa-light-chain-enhancer of activated B cells (NF-κB) and extracellular signal-regulated protein kinase (ERK), which play an important regulatory role in cell proliferation [54]. It has been shown that in the human epidermal growth factor receptor 2 (HER2)/neu-overexpressing breast cancer cells, emodin suppresses the activity of HER-2/neu tyrosine kinase [55]; whereas, in colon cancer cells, emodin inhibits phosphorylation of vascular endothelial growth factor (VEGF) [56]. Another antiproliferative mechanism of emodin activity involves induction of DNA damage by the ROS, whose concentration is considerably increased in the cells treated with this anthraquinone.

#### 2.1.2. Necroptosis

Necroptosis is a kind of programmed cell necrosis, which occurs in response to a specific environmental signal. Morphologically, necroptosis is similar to necrosis. Necrosis is triggered by the receptor-interacting serine/threonine protein kinases receptor-interacting protein 1 (RIP1). Wang et al. [57] demonstrated that emodin induces necroptosis in the cells of renal cancer (RCC), which is resistant to conventional cancer therapy, such as chemotherapy or radiotherapy. In the emodin-treated cells, elevated levels of RIP1, mixed lineage kinase domain like pseudokinase (MLKL) and regulator of fibroblast growth factor 2 (FGF-2) transcription (RFT) were observed. Further studies demonstrated that emodin induces necroptosis through ROS-mediated activation of the c-Jun N-terminal kinases (JNK) signaling pathway and also inhibits glycolysis by downregulation of GLUT1 through ROS-mediated inactivation of the phosphoinositide 3-kinases (PI3K)/AKT signaling pathway.

Similar conclusions were drawn by Zhou et al. [58], who studied the effects of emodin treatment in the U251 glioma cells by targeting the TNF-α/receptor-interacting serine/threonine-protein kinase 1/3 (RIP1/RIP3) signaling pathway. Emodin induced both apoptosis and necroptosis of the cancer cells, and also inhibited proliferation in a dose-dependent manner. The results obtained by molecular methods showed that emodin upregulated the levels of TNF-α, RIP1, RIP3 and MLKL, which confirms induction of necroptosis.

#### 2.1.3. Apoptosis

Emodin is also a strong pro-apoptotic agent. Apoptosis can be initiated through one of two pathways. The first one involves activation of death receptors, including ligands and their receptors, such as FAS, TNF, caspases and B-cell lymphoma 2 (Bcl-2). In the second pathway, pro-apoptotic factors are released from mitochondria as a consequence of mitochondrial DNA damage, growth factor deprivation, hypoxia or activation by oncogenes. In numerous literature reports we can find information that emodin induces apoptosis in various cancer cell lines by its influence on intracellular ROS production and caspase-dependent and mitochondrial signaling pathways.

Trybus et al. [59], proved pro-apoptotic activity of emodin towards HeLa cervical cancer cells; whereas Ma et al. [60] observed that emodin induced apoptosis of fibroblasts obtained from patient with ankylosing spondylitis by increasing active caspase-9, active caspase-3, and bcl-2-like protein 4 (Bax) levels and downregulating Bcl-2.

Other examples are novel compounds targeting cancer cells, based on emodin. The study by Li et al. [61] revealed that a bifunctional molecule of β-dihydro-artemisinin-emodin has high antiproliferative activity (suppressing Ki-67 expression), proapoptotic properties (inhibition of G1/S cell cycle progression), and upregulated the expression of caspase-3/8/9 and Bax, downregulated the expression of Bcl-2 and inhibited HepG-2 cell migration.

#### 2.1.4. Angiogenesis and Metastasis

Angiogenesis is a crucial step in cancer development. The factors that control angiogenesis include transforming growth factor alpha (TGF-α), vascular endothelial growth factor (VEGF) and FGF. It has been demonstrated that emodin inhibited VEGF-A-dependent proliferation, migration, invasion and tube formation of human umbilical vein endothelial cells (HUVEC) in vitro. The research results confirmed that emodin induces HUVEC cell growth arrest in the G_0_/G_1_ phase of the cell cycle by inhibiting cyclin D1 and E expression. An important role in VEGF-A-induced proliferation, migration and differentiation of endothelial cells is played by its receptor KDR/FLK-1 (kinase insert domain-containing receptor). In this case emodin effectively blocks phosphorylation of this receptor and further steps of this signaling pathway, including focal adhesion kinase (FAK), ERK1/2, p38, MAPK and oncogenic protein kinase B (AKT) signaling molecules. Anti-angiogenic activity of emodin is also associated with its ability to induce apoptosis through the p53-dependent pathway [62], whereas overexpression of p53 and interaction of this protein with kinase signaling may contribute to inhibition of VEGF synthesis in cancer cells [63]. Emodin effectively inhibits the expression of the angiogenic-related NF-kB factor, as well as its regulatory factors, including VEGF, MMP-2, MMP-9. Matrix metalloproteinases (MMPs) and CXCR4/CXCR12 chemokine receptors are involved in tumor cell proliferation, invasion and metastasis. Emodin plays a regulatory function in their expression and activity, which was proved for several cancer cell lines, including human tongue cancer SCC-4 cells [64].

The newest study revealed that emodin inhibits breast cancer lung metastasis [65]. Combinatorial therapy with emodin and thymoquinone was also efficient in attenuating migration of MCF-7 breast cancer cells, inducing apoptosis, inhibiting cell proliferation, while enhancing cytotoxicity [66].

### 2.2. Effects of Emodin on the Activity of Drugs

Cancer cells can quickly become resistant to cyto-statics; therefore there is need for new solutions to support cancer therapy. One such method is administration of a cytostatic drug along with a biological substance to support its activity. For example, combination therapy using emodin and doxorubicin sensitize breast cancer cells to doxorubicin by inhibition of proliferation in the DNA damage pathway (decreasing the levels of X-ray repair cross complementing 1 (XRCC1), poly(ADP-ribose) polymerase 1 (PARP1) and RAD51 proteins) and by downregulation of the expression of AKT1 and enhancement of the expression of p53 [67].

Peng et al. [68] determined the effect of emodin on sensitization of lung cancer cells (A549, H460) to cisplatin. It was demonstrated that emodin downregulated Pgp expression, and enhanced cisplatin-induced apoptosis and DNA damage in the cancer cells; however, it had no influence on multidrug resistance-associated protein 1 (MRP1) protein expression, which is associated with multidrug resistance.

Because the gallbladder carcinoma is resistant to many cyto-statics, Wang et al. [69] studied the effect of emodin in combination with cisplatin, carboplatin or oxaliplatin in treating the gallbladder carcinoma cell line SGC996. The proposed drug combination resulted in enhancement of cancer cell sensitivity, along with reduction of glutathione level and inhibition of the expression of MRP1, responsible for multidrug resistance. An in vivo study with tumor-bearing rodents confirmed that cisplatin/emodin co-treatment is beneficial.

Additionally, it has been shown that emodin reverses resistance of cancer cell lines to cyto-statics, for example the resistance of pancreatic cancer cells to gemcitabine [70] or leukemia multidrug resistance by inhibition of P-glycoprotein expression [71].

One of the drugs used for the treatment of advanced kidney cancer and hepatocellular carcinoma (HCC) is sorafenib—an organic compound which is a multi-kinase inhibitor. Kim et al. [72] demonstrated that emodin enhanced the anticancer activity of sorafenib in HepG2, Hep3B, Huh7, SK-HEP-1, and PLC/PRF5 cancer cells. The mechanism of this activity is based on suppressing the cholesterol biosynthesis (inhibition the activity of SREBP-2 protein) and inhibiting the expression of the AKT.

In addition, the study by Chen et al. [73] showed, that emodin enhances anticancer activity of paclitaxel to human NSCLC cells A549. It was found that emodin significantly enhanced PTX-induced apoptosis in A549 cells via increasing the expressions of Bax and active caspase 3 and decreasing the levels of Bcl-2, p-Akt and p-ERK without significant side effects in vivo.

As already mentioned, emodin is used as a substance that supports treatment with other chemotherapeutics. Good effects of such a therapy were noticed, among others, in combination therapy with mitomycin C in lung cancer cells, where inactivation of kinases ERK1/2 and mammalian Rad51 recombination protein associated with the mechanisms of DNA repair was observed. Similarly to the co-treatment with cisplatin, in the case of non-small-cell lung carcinoma (NSCLC) a considerable decrease in the expression of endonucleases was observed. Endonucleases are the enzymes involved in DNA repair by excision of incorrect nucleotides (ERCC1), whereas the combination of emodin with capecitabine induces cytotoxicity by inhibiting the expression of two proteins Rad51 and ERCC1 [74,75,76]. Moreover, emodin reduces resistance of chronic myeloid leukemia cells to imatinib [77].

In addition, emodin combined with berberine significantly inhibited the activity of salt-inducible kinases 3 (SIK3), belonging to the AMPK-related kinases, which elevated expressions in breast cancer cells contributing to tumorigenesis. The combination treatment with emodin and berberin inhibited glycolysis, which attenuated the AKT signaling pathway and led to the cell-cycle arrest [78].

According to studies by Lee et al. [79] emodin inhibited oxidative liver injury via the 5′-adenosine monophosphate-activated protein kinase (AMPK)/yes-associated protein 1 (YAP1) mediated pathway. In vitro, emodin inhibited cell death induced by arachidonic acid + iron, maximally at a dose of 10 μM (EC_50_ > 3 μM). In addition, emodin attenuated the decrease in anti-apoptotic proteins, and restored mitochondrial membrane potential as mediated by the liver kinase B1 (LKB1)-AMPK pathway. In vivo, oral administration of emodin (10 and 30 m/kg, 3 days) decreased acetaminophen (APAP)-induced hepatic damage in mice.

### 2.3. Anti-Inflammatory Activity of Emodin

Anti-inflammatory activity of emodin is widely studied in many disease entities. Emodin is an effective inhibitor of inflammatory markers, which play a pivotal role in cancer development, such as nuclear factor kappa B (NF-ĸB), tumor necrosis factor (TNF-α), interleukines (IL)-1β, IL-6, IL-8, chemokine receptor C-X-C motif chemokine receptor 4 (CXCR4), adhesion molecules intercellular adhesion molecule-1 (ICAM-1), vascular cell adhesion molecule 1 (VCAM-1), endothelial leucocyte adhesion molecule-1 (ELAM-1), and angiogenic factors, such like VEGF. Potential role of emodin in treatment of various inflammatory diseases is based on inhibiting TNF-induced activation of NF-κB, which takes part in transcription of pro-inflammatory genes involved in progression of the disease. It has been confirmed that emodin has therapeutic potential for the treatment of various kinds of inflammation, such as pancreatitis, asthma, arthritis, atherosclerosis, myocarditis, glomerulonephritis and Alzheimer’s disease.

### 2.4. Pancreatitis

Emodin for a long time has been used in the treatment of acute pancreatitis. It alleviates inflammation and reduces pancreas edema. Emodin downregulates the levels of pro-inflammatory cytokines TNF-α and IL-6 and reduces pancreatic paracellular permeability by promoting the expression of the transmembrane proteins of tight junctions: claudin-5 and occludin. These proteins play an important role in the intercellular barriers in epithelium and endothelium. Loss of these functions is associated with acute pancreatitis [80]. Additionally, it has been shown that emodin prevents coagulation and micro-thrombosis, promoting pancreatic cyto-protection by inhibiting the release of pro-inflammatory cytokines and abnormal metabolism of eicosanoids [81]. Moreover, it regulates cellular growth and differentiation, promotes the synthesis of extracellular matrix components, stimulates synthesis of DNA and proteins, and thus plays an important role in pancreatic cell repair and renovation. In this case, the activity of emodin is directly related to its ability to decrease serum amylase level and to increase the expression of the cytokine transforming growth factor beta 1 (TGFβ1) gene [82]. According to literature reports, there are also other mechanisms of action, such as inhibiting the activity of pancreatic myeloperoxidase (MPO) and downregulating the expression of stromal derived factor-1 (SDF-1) [83], inhibiting the NF-κB signaling pathway, and decreasing the content of intestinal inflammatory cytokines TNF-α and IL-1β [84]. It was also observed that emodin reduced the levels of serum amylase and lipase in rats with severe acute pancreatitis (SAP) in a dose-dependent manner and inhibited the P2 × 7/NLR P3 signaling pathway [85]. Emodin administered intra-gastrically at a dose of 40 mg/kg to rats with acute pancreatitis regulated the expression of the autophagy-associated proteins LC3 (B/A) and p62 [86].

Gao et al. [87] provided evidence for the protective activity of emodin in complications associated with acute pancreatitis. Emodin inhibited the production of proinflammatory cytokines, downregulated Nod-like receptor family pyrin domain containing 3 (NLRP3), apoptosis-associated speck-like protein containing a caspase recruitment domain (ASC) and caspase-1 expressions and suppressed NF-κB nuclear accumulation in the lung. In addition, emodin increased nuclear factor erythroid 2-related factor 2 (Nrf2) nuclear translocation and upregulated heme oxygenase-1 (HO-1) expression.

Similarly, Yao et al. [88] studied the effects of emodin on severe acute pancreatitis (SAP) in the rat model. The results of the study showed that emodin alleviates SAP. Furthermore, treatment with emodin markedly inhibited nuclear factor NF-κB DNA binding activity and the serum expression levels of TNFα, interleukin IL-6 and IL-1β. Emodin also decreased malondialdehyde and increased superoxide dismutase levels, therefore its activity may be due to antioxidant mechanisms, which downregulate the expression of the mentioned above cytokines.

According to the most recent research, emodin may be an alternative to dexamethasone for the treatment of severe acute pancreatitis-associated acute lung injury (SAP-ALI). It has influence on the gene expression profile, associated with regulation of mRNA and lncRNA levels in rats treated with emodin and dexamethasone [89].

Tan et al. [90] evaluated the effect of emodin on intestinal dysfunction in rats caused by taurocholate-induced acute necrotizing pancreatitis. Emodin was administered intravenously at a dose of 50 mg/kg, 2 h before the induction of the disease at an interval of 8 h. After 24 h reduction of miR-218a-5p expression level in the intestinal tract was observed, along with an increased expression level of Notch1 and Bcl-2, and decreased levels of RhoA, Rho-associated coiled-coil kinase (ROCK)-1, AKT, Bax, type-II transmembrane protein (FasL), caspase-3, and caspase-9 expression. Emodin inhibited the intestinal cell apoptosis caused by acute severe pancreatitis. The results of Western blot analysis also showed increased expression of proteins in the intestinal tract: occludin, zonula occludens-1 (ZO-1), and E-cadherin. Therefore, emodin can significantly inhibit intestinal cell apoptosis and improve the intestinal dysfunction induced by acute severe pancreatitis

There is also evidence that herbal decoctions containing emodin and other active substances, such as baicalein, renin or chrysin, have an effect on suppressing the expression of mRNA and toll like receptor 4 (TLR4)/NLRP3 proteins associated with the pro-inflammatory signaling pathway, which ameliorates acute pancreatitis [91].

### 2.5. Arthritis

Arthritis is a chronic disorder of joints, which is caused by inflammation of various origins (gout, osteoarthritis, rheumatoid arthritis). In patients with rheumatoid arthritis the most important pathological change is formation of new blood vessels in the synovial membrane, changed by the inflammatory process. The molecular basis of the angiogenesis may be of various kinds: induced by cytokines, VEGF or hypoxia-inducible factor 1-alpha (HIF-1α). It has been proved that emodin used under hypoxic conditions markedly inhibited production of proinflammatory cytokines (TNF-α, IL-6, IL-8), inflammatory mediators (prostaglandin E2) and matrix metalloproteinases (MMP-1 and MMP-13) in IL-1β and LPS-treated synoviocytes. Therapeutic properties of emodin in the treatment of joint inflammation are also associated with inhibition of expression of VEGF, cyclooxygenase 2 (COX-2), hypoxia-inducible factor 1 (HIF-1)α and histone deacetylase (HDAC).

Rheumatoid arthritis (RA) is a systemic disorder of connective tissue, mediated by a number of cytokines and MMPs. Expression of these proinflammatory mediators is controlled by NF-κB. Emodin inhibited the nuclear translocation and DNA binding of NF-κB subunits, which were correlated with its inhibitory effect on cytoplasmic nuclear factor of kappa light polypeptide gene enhancer in B-cells inhibitor alpha (IκBα) degradation in collagen-induced arthritis (CIA) mice. These events further suppressed chemokine production and MMP expression. In addition, emodin inhibited the osteoclast differentiation induced by macrophage colony-stimulating factor (M-CSF) and receptor activation of NF-κB ligand in bone marrow macrophages [92]. Another natural compound of the anthraquinone family that is widely studied with respect to osteoarthritis treatment is aloin [93]—a glycoside derivative of emodin, known also by the name of barbaloin.

Hu et al. [94] in their in vitro and in vivo studies confirmed the protective effect of emodin in the treatment of osteoarthritis. Tissue expression of MMP-12, ADAMTS-4 (a disintegrin and metalloproteinase with thrombospondin motifs 4) and iNOS decreased after the treatment with emodin, as well as COX-2 and PGE2 levels in blood. Efficiency of emodin in vivo at a dose of 80 mg/kg was equivalent to celecoxib, which is a nonsteroidal anti-inflammatory drug and selective COX-2 inhibitor. The effective concentration of emodin for the treatment of chondrocytes was 5 µmol/L.

### 2.6. Alzheimer’s Disease

Alzheimer’s disease is a severe neurodegenerative disease, associated with pathological accumulation of β-amyloid (Aβ) in the brain and misfolding, and aggregation of tau protein, which leads to formation of intracellular paired helical filaments (PHFs) and neurofibrillary tangles. Emodin effectively prevents abnormal aggregation of tau proteins in PHFs [95]. Similarly, Sun and Liu [96] provided evidence that that emodin may be used in prevention and treatment of Alzheimer’s disease.

One of the risk factors for different human disorders, including neurodegenerative diseases such as Alzheimer’s or Parkinson’s diseases, is elevated level of homocysteine in blood (hyper-homocysteinemia). According to Zeng et al. [97] emodin administered to rats with hyper-homocysteinemia-induced cognitive deficits (HCY-E0) at a dose of 80 mg/kg/day decreased the levels of β-amyloid and tau phosphorylation. Moreover, in the hippocampi of the rats the neuron numbers and levels of synaptic proteins increased. It was also observed that hyper-homocysteinemia-induced microangiopathic alterations, oxidative stress and elevated DNA methyltransferases 1 were ameliorated.

According to the other research team [98], emodin improved cognitive functions by activating the protein kinase C signaling pathway (PKC), attenuating oxidative stress and inflammatory response in mice with Alzheimer’s disease.

Li et al. [99] studied potential neuroprotective effects associated with the antioxidant activity of emodin in U251 cells that were subjected to β-amyloid peptide (Aβ)-induced apoptosis and in amyloid precursor protein (APP)/presenilin-1 (PS1) double-transgenic mice. In apoptotic U251 cells, 3-h emodin pretreatment prior to 24-h Aβ co-exposure improved cell viability, suppressed lactate dehydrogenase leakage and caspase-3, -8 and -9 activation to inhibit apoptosis. An 8-week treatment of mice with emodin improved spatial memory and learning ability and decreased anxiety. It was also found that emodin regulated Nrf2 pathway and decreased Aβ deposition. It has a neuroprotective effect on human astroglioma cell line U251.

Because of the well-proven activity of emodin, Paranjape et al. [100] studied the ability of secondary metabolites of *Aspergillus nidulans* to inhibit tau aggregation. The results revealed that a hydroxyl derivative of emodin, 2,ω-dihydroxyemodin is a stronger tau aggregation inhibitor than emodin.

### 2.7. Antimicrobial Activity

According to current research, the biological activity of emodin, including its antimicrobial properties, depends on its interactions with components of biological membranes [101,102].

Shifa et al. [103] isolated two anthraquinones: emodin and chrysophanol from the roots of *Rumex abyssinicus,* which were used to cure various diseases, including gonorrhea, typhus and hepatitis. The two compounds were next tested for antimicrobial activity. Zones of growth inhibition were measured for such pathogens as: *S. aureus* ATCC25903, *K. pneumoniae* NCTC13368, *E. coli* ATCC 25722, *P. aeruginosa* DSMZ 1117 and *C. albicans* and *S. cerevisiae*, using concentrations of 100 mg/mL and the agar disc diffusion method. In the case of emodin, the highest activity was observed for the gram-positive bacteria of *S. aureus* (the growth inhibition zone 23 mm). Weaker activity was observed for the gram-negative strains *P. aeruginosa* and *E. coli*, for which the measured growth inhibition zone was 18 mm. Both emodin and chrysophanol were inactive to the yeasts *S. cerevisiae*.

Emodin also was active towards methicillin-resistant *S. aureus* (MRSA), where the MIC value was 4 µg/mL. An iodine atom in the aromatic ring (2-iodoemodin and 2,4-diiodoemodin) increased the antibacterial activity (MIC equal to 1–2 µg/mL), whereas chlorine (2,4-dichloroemodin) and bromine (2,4-dibromoemodin) substitution maintained the activity, with higher cytotoxic activity observed against Vero cells (IC_50_ in the range 9.7–18.7 µM).

Similar conclusions were reached by Promgool et al. [104], who established the MIC values for emodin towards the gram-positive strains *Bacillus* *cereus* TISTR 687, methicillin resistant *S. aureus* (MRSA)-SK1 and *S. aureus* TISTR 1466. The established MIC values for the gram-positive bacteria were 16, 4 and 16 µg/mL, respectively, whereas, for the gram-negative bacteria *P. aeruginosa* TISTR 781 and *Salmonella typhimurium* TISTR780, the measured MIC value was 128 µg/mL.

Furthermore, the minimal inhibitory concentration (MIC) and minimal bactericidal concentration (MBC) values for emodin against *Haemophilus parasuis*, which is the causative agent of Glässer’s disease, were 32 and 64 μg/mL, respectively [105]. Emodin was isolated from *P. cuspidatum*.

According to the Chukwujekwu et al. [106], studies, the minimum inhibitory concentration (MIC) values of emodin isolated from the roots of *Cassia occidentalis* against *Bacillus subtilis* and *S. aureus* were 7.8 × 10^−3^ and 3.9 × 10^−3^ mg/mL, respectively. Emodin was not active against two gram-negative bacteria: *Klebsiella pneumoniae* and *E. coli*, even at the highest tested concentration (5.0 × 10^−3^ mg/mL).

Emodin also exhibited antifungal activity against *C. albicans* with the inhibition zone of 1.1 mm (76%) at the concentration of 5 mM, while against *A. fumigatus* AF293 it was inactive. It also showed antimicrobial activity against pathogenic trichomonads *T. foetus*. The IC_50_ value was about 8 µM with the 48% growth inhibition at the designated concentration, whereas at a concentration of 10 µM emodin was inactive to *S. enterica*, *L. monocytogenes*, *S. aureus*, *B. cereus*, *E. coli* K12, *L. acidophilus*, *L. rhamnosus* GG and *L. reuteri* [107].

According to the studies by Xu et al. [108], emodin may be a novel pharmacological agent for the prevention and treatment of dental caries. The growth and acid production of *S. mutans* were significantly inhibited by emodin at the concentration of 0.5–2 mg/mL. Emodin also significantly suppressed the synthesis of insoluble glucans by *S. mutans*. Furthermore, the topical application of emodin reduced the incidence and severity of carious lesions in rats. Synergistic effect of emodin in combination with ampicillin or oxacillin against methicillin-resistant *S. aureus* was described by Lee at al. [109].

It was shown that emodin may significantly reduce the damage to the intestinal epithelial barrier in sepsis, inhibit intestinal barrier permeability and protect intestinal barrier integrity, by elevating expression levels of the TJ proteins: claudin-3, ZO-1 and occludin in cecal ligation and puncture (CLP) rats [110].

### 2.8. Antiallergic Activity

Emodin belongs to the group of natural substances with antiallergic properties. Wang et al. [111] explored the inhibitory effects of emodin on the IgE-mediated allergic response in rat basophilic leukemia (RBL-2H3) cells by measuring the release of granules and cytokines. The results of these studies indicate that emodin has inhibitory effect on β-hexosaminidase activity and TNF-α with IC_50_ 5.5 µM and 11.5 µM respectively. These factors, along with IL-4, indicate development of late-phase allergic reaction after exposure to an allergen. A significant inhibiting effect of emodin to β-hexosaminidase activity suggests that this compound may be a promising antihistaminic agent.

Another research group provided evidence that emodin inhibits production of eicosanoids and leukotrienes, as well as secretion of cytokines TNF-α and IL-6 in IgE/Ag-stimulated mast cells [112].

## 3. Metabolism of Emodin

Cytochromes are a group of enzymes with monooxygenase activity which are widespread in almost all tissues of the body, with the highest activity in the liver. Mueller et al. [113] studied the metabolism of emodin by cytochrome P450 enzymes. They observed two emodin metabolites, ω-hydroxy-emodin and 2-hydroxy-emodin, which were formed at the rate depending on the inductor (e.g., 3-methylcholanthrene, α-naphthoflavone). The anthraquinone chrysophanol (1,8-dihydroxy-3-methylanthraquinone) is transformed, in a cytochrome P450-dependent oxidation, to aloe-emodin (1,8-dihydroxy-3-hydroxymethylanthraquinone) as the major product. The obtained metabolites were tested for antitumor activity using in vitro micronucleus test. It was observed that 2-hydroxyemodin induced higher micronucleus frequencies in mouse lymphoma L5178Y cells than emodin.

There are also known transformations of emodin using transgenic crown galls of *Panax quinquefolium*. The following products were observed: beta-glucoside [emodin-6-*O*-beta-D-glucopyranoside (yield 19.2%) and a hydroxylated derivative—citreorosein (yield 54.6%) [114].

Ghimire et al. [115] used glycosyltransferase from *Bacillus licheniformis* DSM13 (YjiC) expressed in *E. coli* cells for enzymatic modification of emodin in vitro and in vivo. Emodin-*O*-β-D-glucoside was the major product of this transformation. The biological tests demonstrated that glycosylation of anthraquinones enhances their aqueous solubility while retaining their biological activities.

The other research group found that cell cultures of *Ajuga reptans* L. converted emodin to five compounds, among which the main were: 6-*O*-malonyl galactoside, 6-*O*-galactoside, dimalonyl-diglycoside, and acetyl-diglycoside [116].

Souginnis et al. [30] evaluated the in vivo toxicity of emodin administered orally and intraperitoneally to mice in doses that have been shown to be efficient in cancer studies: 20 mg/kg, 40 mg/kg and 80 mg/kg, applied for 12 weeks. The levels of glucuronidated emodin in the blood of mice were measured. It appeared that female mice metabolized emodin at a faster rate than male mice. The results showed that emodin is safe for use. Neither of the doses caused any pathophysiological perturbations in the organs.

## 4. Conclusions

Emodin is a natural anthraquinone with a wide range of therapeutic activities. Determination of the scope of its anticancer activity on the molecular level is extensively studied globally. Based on recent reports, emodin is a strong inhibitor of kinases, such as Her-2/neu, CK2 and PKC, and has regulatory function in signaling pathways NF-κB, STAT3, AKT, MMP and Bax/Bcl-2. Moreover, emodin combined with other chemotherapeutics and in targeted therapies suppresses growth of cancer cells. It prevents development of neurodegenerative and inflammatory processes and inhibits growth of microbial pathogens that cause infectious diseases in people. Nevertheless, for the safe use of emodin in combination with other commonly used drugs, detailed clinical studies are needed, because of the uncertainty about toxicity of such multicomponent therapeutics. It is also important to develop new forms of delivery of anthraquinones, including emodin, with the help of natural, biodegradable and non-toxic nano-transporters.

## Data Availability

All data are publicly available.
